# A methodological framework and experimental protocol for proactive human–robot collaboration with multimodal intention prediction and adaptive control

**DOI:** 10.3389/frobt.2026.1825254

**Published:** 2026-05-07

**Authors:** Juan Escobar-Naranjo, Carlos García-Ávila, Ivón O. Benítez-González, Paúl Baldeón-Egas, Wilmer Albarracín-Guarochico

**Affiliations:** 1 Faculty of Systems, Electronics and Industrial Engineering, Technical University of Ambato, Ambato, Ecuador; 2 Faculty of Engineering, Andrés Bello Catholic University, Montalbán, Venezuela; 3 Production Department, Electric Corporation of Ecuador, Cuenca, Ecuador; 4 Higher School of Engineering and Technology, International University of La Rioja, Logroño, Spain; 5 Department of Engineering Sciences, Israel Technological University, Quito, Ecuador

**Keywords:** adaptive control, deep learning, human–robot collaboration, industry 5.0, intention prediction, methodological framework, multimodal perception

## Abstract

Industry 5.0 requires collaborative robots that can anticipate operator needs to improve fluency and safety in assembly. However, many human–robot collaboration (HRC) systems still treat perception, intention inference, and control as separate components. This study presents a theoretical perception–cognition–action framework that explicitly couples multimodal intention prediction with proactive and adaptive control. Multimodal observations such as RGB-D vision, gaze, wrist force/torque, robot joint state, and previous robot action are encoded by a hybrid Convolutional Neural Network (CNN)– Long Short-Term Memory (LSTM)–Transformer to estimate (i) a probability distribution over future human intentions and (ii) a short-horizon motion trajectory, trained with a composite loss that jointly optimizes classification and regression with kinematic coherence. The predicted intention probability is embedded into an augmented Markov Decision Process state, enabling a Soft Actor–Critic agent to learn continuous policies with rewards designed for synergy, efficiency, safety, and fluency. The main contributions of this study are the formal probabilistic linkage from intention prediction to adaptive control, the definition of a multi-output cognitive objective, and the design of an implementation-ready experimental protocol for future empirical validation. Overall, the proposed methodological framework and experimental protocol provide a reproducible basis for future empirical validation of proactive human–robot collaboration in industrial assembly tasks.

## 1 Introduction

**FIGURE 1:**
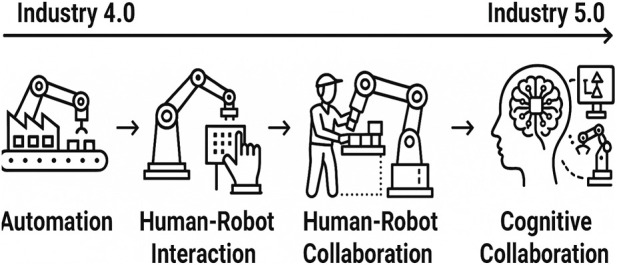
Evolution from Industry 4.0 to 5.0: from reactive to proactive cobots.

The transition from Industry 4.0 to Industry 5.0 has brought a paradigmatic shift in manufacturing, redirecting the focus from pure automation to human-centered symbiosis (Cohen et al., 2025; Pietrantoni et al., 2024; Terras et al., 2025). Recent Industry 5.0 discussions have further emphasized human-centric manufacturing supported by Human Digital Twins, which can represent operator capabilities and constraints to personalize robotic assistance while preserving wellbeing (Bucci et al., 2024). The conceptual evolution of these industrial revolutions is summarized in Figure 1.

In this new context, collaborative robots cease to be mere tools and become active partners in the production processes. They are designed to augment the operator’s cognitive and physical capabilities, improving the fluency, safety, and efficiency of shared assembly tasks (Cohen et al., 2025; Terras et al., 2025). The success of this symbiosis depends on the robot’s ability to interact in an intuitive, safe, and efficient manner, which in turn requires a deep understanding of the environment and, fundamentally, the human partner’s state and intentions (Cohen et al., 2025; Pietrantoni et al., 2024; Terras et al., 2025). However, most current collaborative systems operate reactively. They typically lack genuine cognitive understanding and are frequently driven by predefined task scripts and conservative, stop-and-go safety behaviors. Such limited adaptability can create efficiency bottlenecks and may reduce human predictability and trust in collaboration Karbouj et al., 2024).

Recent literature has addressed this challenge by integrating deep learning and multimodal perception. Various studies have explored single sensory channels to infer intention, including computer vision (Mendez et al., 2024), gesture recognition (Baptista et al., 2024), gaze tracking as a predictor of physical actions (Belcamino et al., 2024; Di Stefano et al., 2025), and physiological signals such as electroencephalography (EEG) or electromyography (EMG) (Abidi, 2024). Temporal models, including recurrent neural networks (LSTM) and Transformers, have proven promising for capturing the spatiotemporal dynamics of human motion (Zhu et al., 2025; Zhou et al., 2024). In physical human–robot interaction, comparative studies have also evaluated CNN versus LSTM baselines for intention prediction, highlighting the benefits of explicitly modeling temporal dependencies when interactions unfold over time (Zadeh et al., 2025). In the control domain, Deep Reinforcement Learning (DRL) has emerged as a robust solution for developing adaptive control policies in real time (Han et al., 2023; Jeon et al., 2024).

Despite these advances, there are still significant gaps. First, although context- and intention-aware human motion prediction has recently gained traction (Gómez-Izquierdo et al., 2025), task context and human–object interactions are still not consistently represented in a way that can be directly exploited by downstream decision-making and control in assembly settings. Second, there is a gap between intention prediction and control execution: predictive models are often not smoothly integrated with adaptive controllers, or DRL controllers struggle to generalize when transferred from simulation to reality (Yuan et al., 2022). Third, emerging vision–language architectures may exhibit inference latency that is excessively high for real-time control (Kawaharazuka et al., 2025). Finally, collecting multimodal interaction data remains challenging, motivating exploration in Virtual Reality (VR) environments to generate robust datasets (Maddipatla et al., 2025).

Moreover, hybrid CNN–LSTM–Transformer pipelines have been explored in other sequential prediction domains, reinforcing their practicality in combining local feature extraction, temporal memory, and long-range attention (Wang et al., 2025). Although recent frameworks incorporate multimodal perception or intention inference, most operate as sequential pipelines, in which predicted intentions inform heuristic or reactive behaviors. As highlighted by recent systematic reviews, there is still no unified formulation in which intention prediction is embedded as a probabilistic latent variable within the control state itself, enabling formally proactive decision-making rather than *post hoc* adaptation (Semeraro et al., 2023; Kedia et al., 2024; Cai et al., 2026). However, a unified framework that formally couples multimodal intention prediction with proactive adaptive control within a single perception–cognition–action loop is lacking. In Table 1 a comparison between the different modalities of the most representative approaches is presented.

**TABLE 1:** Comparison with representative state-of-the-art approaches in HRC.

Work	Modalities	Intention prediction	Motion prediction	Control strategy
Zhou et al. (2024)	Vision	No	Yes	Rule-based
Di Stefano et al. (2025)	Gaze	Yes	No	Reactive
Kedia et al. (2024)	Multimodal	Yes	No	Learned
This work	Multimodal + robot action	Yes	Yes	Proactive DRL

To address these limitations, this study proposes a Multimodal Deep Learning Framework for Intention Prediction and Adaptive Control in Collaborative Assembly Robotics. Our proposal integrates and mathematically formalizes the synergy among a multimodal perception module, hybrid cognitive model such as CNN–LSTM–Transformer, and an adaptive control module based on DRL (Soft Actor–Critic). The main contributions of this methodological proposal are as follows:

1. The design of a hybrid deep learning architecture for intention prediction that explicitly integrates assembly task context, human–object interactions and feedback from robot actions.
2. The mathematical formalization of a unified framework that bridges cognition and action by modeling the task as a Markov Decision Process, where the DRL agent’s state vector is directly augmented with the probabilistic output of the intention prediction model.
3. The design of a multi-output loss function for the cognitive model that simultaneously optimizes intention classification and future trajectory regression (losses $L_{\mathrm{int}}$ , $L_{\mathrm{mov}}$ , and $L_{va}$ ).
4. The reward function for the Deep Reinforcement Learning agent using the Soft Actor–Critic algorithm is designed to optimize synergy and proactivity by minimizing human idle time, enhance fluency by reducing motion jerk, and ensure efficiency and safety.
5. The design of a rigorous, implementation-ready experimental validation protocol that compares the proposed framework against reactive baselines using objective and subjective metrics such as the NASA Task Load Index (NASA-TLX) questionnaire (Hart and Staveland, 1988).

Recent research indicates that progress toward truly proactive collaborative robotics requires the integration of three technological pillars: multimodal perception, intention prediction models, and adaptive control based on deep reinforcement learning. At the system level, multimodal sensor fusion deployed at the edge has been shown to reduce latency and improve the robustness of collaborative robotic arms under realistic operational disturbances (Feng, 2025). Regarding perception, the literature aims to enable the fusion of vision, gaze tracking, and force signals to significantly enhance a system’s ability to infer human states in real time (Mendez et al., 2024; Baptista et al., 2024; Belcamino et al., 2024; Zhao. 2025). In parallel, hybrid CNN–LSTM models and, more recently, transformer-based architectures have shown strong performance in capturing spatiotemporal dependencies and anticipating human actions (Zhou et al., 2024; Laplaza et al., 2025). In the control domain, approaches grounded in Deep Reinforcement Learning, particularly Actor–Critic algorithms such as Soft Actor–Critic (SAC), demonstrate strong performance in learning adaptive and robust policies for robot manipulation and human–robot collaboration (Husakovic et al., 2025). Nevertheless, the literature also highlights a critical gap: these components are often developed in isolation, without a unified formulation that explicitly links intention prediction to robotic control, thereby limiting genuine proactivity in assembly tasks (Kedia et al., 2024). This conceptual and methodological gap motivates the framework proposed in this study, which integrates multimodal perception, cognitive intention prediction, and adaptive control within a unified perception–cognition–action cycle.

## 2 Materials and methods

This section details the methodological core of the proposal, formalizing the system architecture, multimodal state representation, cognitive predictive model, and proactive adaptive controller.

### 2.1 System architecture and general probabilistic formulation

**FIGURE 2:**
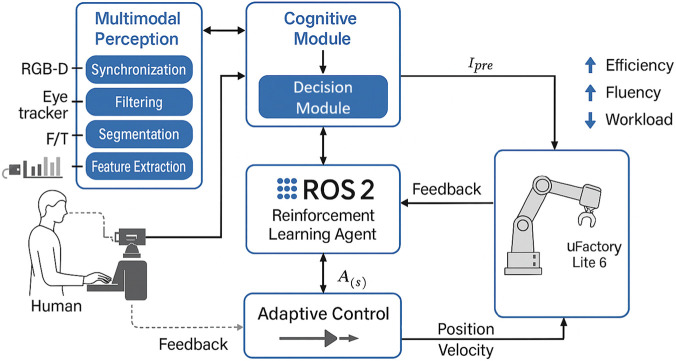
General architecture of the proposed framework.

The framework is based on a perception–cognition–action loop composed of two components operating in tandem: the Cognitive Module (CM) and the Decision Module (DM). As shown in Figure 2, the proposed framework integrates multimodal sensing, a hybrid cognitive model, and a DRL-based controller within a unified perception–cognition–action cycle to achieve this.

1. Cognitive Module (CM): At each time step t , the CM observes the recent history of multimodal observations $\left(H_{t}=O_{t-n},\ldots ,O_{t}\right)$ and estimates a probability distribution over the operator’s future intentions:

$$I_{\mathrm{pred}}=P\left(I_{h}\left(t+k\right)|H_{t}\right),$$ (1)

where k represents the prediction horizon.

1. Decision Module (DM): The DM is a DRL agent that receives the current system state $S_{t}$ , which includes the intention prediction $I_{\mathrm{pred}}$ from the CM. The DM executes a robotic action $a_{r}\left(t\right)$ according to the learned policy π .

The general probabilistic objective is to find the optimal robotic policy π∗ that maximizes the expected cumulative reward R . The optimal policy should be conditioned not only on the observable physical state $S_{t}$ but also on the predicted future human intention $I_{\mathrm{pred}}$ :

$$\pi *\left(a_{r}\left(t\right)\right)=\pi \left(a_{r}\left(t\right)|S_{t},I_{\mathrm{pred}}\right),$$ (2)

This formulation departs from prior intention-aware HRC approaches, where intention is typically used as a contextual cue or a heuristic trigger. By explicitly embedding the predicted intention distribution into the Markov Decision Process (MDP) state, the proposed framework enables the policy to differentiate between physically identical states with distinct future human intents, a capability that has been identified as missing in recent intention-aware control architectures (Kedia et al., 2024; Gómez-Izquierdo et al., 2025; Laplaza et al., 2025).

### 2.2 Multimodal perception module: definition of the observation $O_{t}$

At each time step t , the system captures a multimodal observation tuple $O_{t}$ , which is defined as:

$$O_{t}=\left(V_{t},G_{t},F_{t},J_{t},A_{r,t-1}\right),$$ (3)

where the components are listed in Table 2. The inclusion of $A_{r,t-1}$ (the previous robot action) is essential, as it enables the cognitive model to learn the interdependence between robot actions and human intentions, addressing the gap identified by Kedia et al. (2024).

**TABLE 2:** Definition of the multimodal observation vector $O_{t}$

Symbol	Sensor	Dimensionality	Description
$V_{t}$	RGB-D Camera	H∗W∗4	RGB-D image of the scene
$G_{t}$	Eye tracker	$R^{2}$	2D coordinates of gaze fixation point
$F_{t}$	Wrist-mounted F/T sensor	$R^{6}$	Force/torque sensor reading
$J_{t}$	Robot encoders (6-DoF)	$R^{12}$	Robot joint state (positions and velocities)
$A_{r,t-1}$	Controller logs	$R^{6}$	Robot action command at time t−1

### 2.3 Hybrid cognitive model for intention prediction

The hybrid CNN–LSTM–Transformer architecture was selected to balance local spatial feature extraction, short-term temporal memory, and long-range dependency modeling while maintaining computational feasibility for real-time robotic control. The model is defined for Intention Prediction $P\left(I_{h}\left(t+k\right)|H_{t}\right)$ , where $H_{t}=\left\{O_{t-n},\ldots ,O_{t}\right\}$ represents the history of observations. $I_{h}$ is a discrete variable denoting a set of predefined intentions, such as $I=\left\{\prime reachpartA^{\prime },\prime installpartA^{\prime },\prime wait^{\prime }\right\}$ .

The CNN branch consists of three convolutional layers with 3×3 kernels and ReLU activation. The LSTM layer uses 256 hidden units to capture temporal dynamics, followed by a Transformer encoder with 8 attention heads and a feed-forward dimension of 512, ensuring long-range dependency modeling.

#### 2.3.1 Feature extraction $x_{t}$

1. Vision-Gaze Fusion. Gaze data $G_{t}$ are used to generate an attention heatmap

$$G_{t}^{\text{map}}\in R^{H\times W\times 1},$$ (4)

modeled as a Gaussian, centered at $G_{t}$ (Asad et al., 2025).

1. Visual Processing. A CNN extracts visual features weighted by gaze-based attention as follows:

$$v_{t}=\text{CNN}\left(\text{Concat}\left(V_{t},G_{t}^{\text{map}}\right)\right),v_{t}\in R^{d_{v}}.$$ (5)

1. Vector Processing. The remaining data were processed using a multilayer perceptron (MLP):

$$u_{t}=\text{MLP}\left(\text{Concat}\left(F_{t},J_{t},A_{r,t-1}\right)\right),u_{t}\in R^{d_{u}}.$$ (6)

1. Multimodal Feature Vector.

$$x_{t}=\text{Concat}\left(v_{t},u_{t}\right),\;x_{t}\in R^{d_{x}}.$$ (7)

#### 2.3.2 Context weighting

A Transformer encoder is applied to the LSTM output to weight the importance of different time steps and capture longer-range dependencies (Zhou et al., 2024; Gómez-Izquierdo et al., 2025). The self-attention mechanism is as follows:

$$Q=H_{\text{lstm}}W_{Q},$$ (8)

$$K=H_{\text{lstm}}W_{K},$$ (9)

$$V=H_{\text{lstm}}W_{V},$$ (10)

$$H_{\text{attn}}=\text{Attention}\left(Q,K,V\right)=\text{softmax}\left(\frac{QK^{⊤}}{\sqrt{d_{k}}}\right)V.$$ (11)

where $W_{Q},W_{K},\text{ and }W_{V}$ are the learnable weight matrices for the Query, Key, and Value projections, respectively, which transform the LSTM output $H_{\mathrm{lstm}}$ into the attention space.

#### 2.3.3 Prediction head - dual output

The sequence feature vector $z_{t}$ obtained from $H_{\text{attn}}$ feeds two prediction heads as follows:

1. Intention Prediction $\left(I_{\text{pred}}\right)$ . A probability distribution over future intentions.

$$\begin{array}{c} I_{\text{pred}}=P\left(I_{h}\left(t+k\right)|H_{t}\right)\\ =\text{softmax}\left({\text{MLP}}_{\text{head\_int}}\left(z_{t}\right)\right). \end{array}$$ (12)

1. Motion Prediction $\left({\hat{x}}_{\text{mov}}\right)$ . An anticipated trajectory, such as hand positions, for the next Δt seconds:

$${\hat{x}}_{\text{mov}}={\text{MLP}}_{\text{head\_mov}}\left(z_{t}\right).$$ (13)

#### 2.3.4 Loss function of the cognitive model

This composite loss encourages the model to not only classify the operator’s future intention correctly but also to generate physically plausible motion forecasts, which is critical for ensuring smooth and safe proactive assistance in collaborative assembly tasks. The cognitive model is trained using a composite loss function $L_{\text{total}}$ that jointly optimizes both outputs:

$$L_{\text{total}}=L_{\text{int}}+\lambda L_{\text{mov}}+\alpha L_{va},$$ (14)

where:

-
$L_{\text{int}}$ (*Intention Loss*) is the categorical cross-entropy between the predicted intention $I_{\text{pred}}$ and the ground-truth label $y_{\text{int}}$ .
-
$L_{\text{mov}}$ (*Motion Loss*) is the mean squared error (MSE) between the predicted trajectory ${\hat{x}}_{\text{mov}}$ and the true trajectory $x_{\text{mov}}$ .
-
$L_{va}$ (*Velocity/Acceleration Loss*) is the MSE over the first and second derivatives of the trajectory (velocity and acceleration), as proposed by Zhou et al. (2024), enforcing temporal coherence and penalizing physically implausible motion.
-
λ and α are hyperparameters weighting the relative importance of each loss term.

The hyperparameters will be initially set to λ=0.5 and α=0.1 as preliminary convergence tests in simulation. These values ensure that the intention classification $\left(L_{\mathrm{int}}\right)$ remains the primary objective while the motion regression terms $\left(L_{\mathrm{mov}},L_{va}\right)$ act as strong regularizers. Final optimization of these parameters will be conducted using a Bayesian Optimization approach to minimize the validation error across all three objectives.

### 2.4 Proactive adaptive control: formulation as an MDP

The Decision Module is formalized as a Markov Decision Process. The key contribution of this study lies in defining the state space, which explicitly integrates intention prediction. The MDP tuple ⟨S,A,P,R⟩ is defined in Table 3.

**TABLE 3:** MDP formulation for proactive collaborative assembly.

Component	Symbol	Formal definition	Description
State space	S	$S_{t}=\left(S_{\text{obs}},S_{\text{task}},I_{\text{pred}}\right)$	Augmented state: Core of the framework. The DRL state includes observation, tasks, and predicted intentions.
$S_{\text{obs}}$		$R^{d_{x}}$	Current multimodal feature vector (output $x_{t}$ from Section 2.3.1).
$S_{\text{task}}$		${\left\{0,1\right\}}^{m}$	Binary vector tracking the progress of the m assembly subtasks.
$I_{\text{pred}}$		I	Probabilistic representation of the predicted human intention.
Action space	A	$A_{t}\in R^{6}$	Continuous action space, Cartesian or joint velocity control for uFactory Lite 6.
Transition function	P	$P\left(S_{t+1}|S_{t},A_{t}\right)$	State transition probability.
Reward function	R	$R\left(S_{t},A_{t}\right)$	Scalar function $r_{t}$ defining the objective of the study.

The inclusion of $I_{\text{pred}}$ in state $S_{t}$ allows the DRL agent to learn a policy $\pi \left(a_{t}|S_{t}\right)$ that distinguishes between physically identical states with different predicted human intentions.

### 2.5 Optimal control policy via soft actor-critic

Soft Actor–Critic is selected over alternative DRL methods like PPO or TD3 due to its robustness in continuous action spaces and entropyregularized exploration, which is particularly suitable for smooth, human-safe motion generation in HRC scenarios. The SAC algorithm (Husakovic et al., 2025) is an off-policy, sample-efficient and designed for continuous control, promoting exploration by maximizing entropy. Compared to movement-primitive-based or policy-gradient approaches frequently adopted in HRC, SAC provides improved robustness under stochastic human behavior owing to its entropy-regularized objective. This property is particularly relevant when intention predictions are uncertain, as it prevents premature policy convergence and supports safer exploration (Han et al., 2023; Husakovic et al., 2025).

#### 2.5.1 Objective function of SAC

SAC seeks a policy π that maximizes both the expected reward and entropy of the policy. The objective function is defined as follows:

$$J\left(\pi \right)=\overset{T}{\underset{t=0}{\sum }}E_{\left(s_{t},a_{t}\right)∼\rho_{\pi }}\left[r\left(s_{t},a_{t}\right)+\alpha H\left(\pi \left(\cdot |s_{t}\right)\right)\right],$$ (15)

where $\rho_{\pi }$ denotes the state-action marginals under policy π , $r\left(s_{t},a_{t}\right)$ is the reward function, $H\left(\pi \left(\cdot |s_{t}\right)\right)$ is the policy entropy, and α is the temperature coefficient that balances reward maximization (exploitation) and entropy (exploration).

#### 2.5.2 Reward function definition

The design of the reward function $r_{t}$ is critical in this regard. The weighting coefficients $w_{\mathrm{task}},w_{\mathrm{safe}},w_{\mathrm{syn}},\text{ and }w_{\mathrm{flu}}$ are scalar values that balance the multi-objective reward structure, allowing the designer to prioritize safety or efficiency depending on the task requirements. A composite reward is proposed as follows:

$$r_{t}=w_{\text{task}}\;r_{\text{task}}+w_{\text{safe}}\;r_{\text{safe}}+w_{\text{syn}}\;r_{\text{syn}}+w_{\text{flu}}\;r_{\text{flu}}+r_{\text{alive}},$$ (16)

where w is the weighting coefficient, and the components are:

1. Task Reward $\left(r_{\text{task}}\right)$ : Rewards efficiency and completion.
  -
$r_{\text{task}}=+R_{\text{subtask}}$ (positive reward) if action $A_{t}$ leads to completion of a subtask (change in $S_{\text{task}}$ ).
  -
$r_{\text{task}}=+R_{\text{final}}$ (large reward) upon task completion.
2. Safety Reward $\left(r_{\text{safe}}\right)$ : Penalizes risk.
  - The minimum human-robot distance $d_{hr}$ is computed.
  - A safety zone $d_{\text{safe}}$ is defined; violation incurs a severe, nonlinear penalty:

$$\begin{array}{c} r_{\text{safe}}=-C_{\text{safe}}\cdot \exp\left(-\frac{d_{hr}-d_{\text{safe}}}{\sigma_{\text{safe}}}\right)\\ \;\text{if }d_{hr}<d_{\text{safe}}. \end{array}$$ (17)

where the penalty magnitude is governed by $C_{\mathrm{safe}}$ and $\sigma_{\mathrm{safe}}$ is the spatial decay constant, which defines the softness of the safety boundary around the operator.

1. Synergy Reward $\left(r_{\text{syn}}\right)$ : Rewards correct proactivity.
  -
$r_{\text{syn}}=+R_{\text{proactive}}$ if the robot initiates an action $A_{t}$ aligned with the predicted intention $I_{\text{pred}}$ and this results in negligible human idle time $t_{\text{idle\_h}}\approx 0$ .
  -
$r_{\text{syn}}=-R_{\text{mistake}}$ if the robot initiates a proactive action aligned with $I_{\text{pred}}$ , but the human’s actual intention is different.
2. Fluency Reward $\left(r_{\text{flu}}\right)$ : Penalizes abrupt or erratic motions.

$$r_{\text{flu}}=-J\left(t\right),$$ (18)

where J(t) is a measure of the robot jerk (time derivative of acceleration). Minimizing the jerk produces smoother and more predictable trajectories.

1. Alive Penalty $\left(r_{\text{alive}}\right)$ : A small constant penalty applied at each time step t while the task remains incomplete, promoting time efficiency:

$$r_{\text{alive}}=-C_{\text{step}}.$$ (19)

**FIGURE 3:**
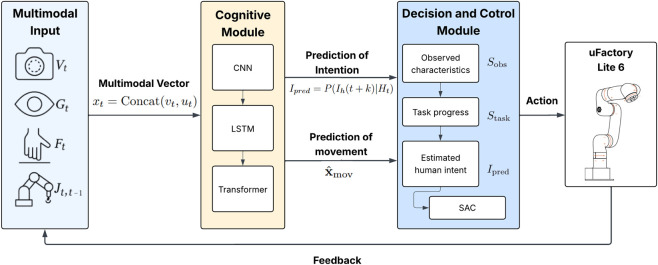
Hybrid CNN–LSTM–Transformer framework and perception–cognition–action loop.

The general information flow of the proposed framework is illustrated in Figure 3, which depicts the multimodal perception input, hybrid deep learning cognitive module, and decision module based on the Soft Actor–critic algorithm forming a unified perception–cognition–action loop.

## 3 Experimental design and validation protocol

This section describes a prospective experimental design and validation protocol intended for implementation in future empirical studies, rather than reporting completed experiments. It also includes the study design, evaluation metrics, and testable hypotheses that will be used to empirically assess the proposed framework against the reactive baselines.

### 3.1 Validation platform and assembly task

This section describes the experimental platform and task configuration intended for the future empirical validation of the proposed framework.

Platform: The experimental cell will consist of the following components:

1. Robot: The uFactory Lite 6 is a 6-DoF robotic arm with a payload of 0.6 kg , a reach of 440 mm , and a repeatability of ±1 mm , ensuring the precision required for delicate assembly tasks (LTD, 2023).
2. Contact Sensor: A wrist-mounted Force/Torque (F/T) sensor.
3. Vision System: An external Intel RealSense D435 RGB-D camera will be used, providing a depth resolution of 1280×720 at 30 fps and a field of view of 85.2°×58° , which is critical for capturing high-fidelity multimodal observations (Corporation, 2018).
4. Gaze Tracking: An eye-tracking device worn by the operator.
5. Software: The system will be integrated using Robot Operating System 2 (ROS 2).

Assembly Task: A standard collaborative assembly task will be employed, following procedures inspired by prior literature.

Role Definition:

- Human Role: Perform assembly operations requiring decision-making.
- Robot Role: Manage inventory, anticipate the part the human will need next, retrieve it, and proactively deliver it.

**FIGURE 4:**
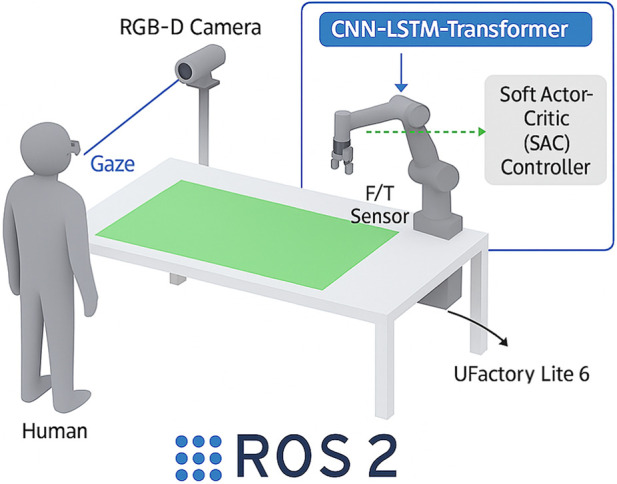
Schematic of workspace.

Figure 4 illustrates the schematic layout of the collaborative assembly workspace used for experimental validation, showing the spatial arrangement of the uFactory Lite 6 robot, human operator, multimodal sensors, and assembly area integrated into the ROS 2 environment.

### 3.2 Detailed experimental protocol design

At the time of writing, this protocol has not yet been executed; however, it is presented to ensure reproducibility, transparency, and rigor for subsequent experimental validation.

- Participants: A total of N=30 participants will be recruited. The inclusion criteria were right-handedness, normal or corrected vision, and no prior experience in human-robot collaboration (HRC).
- Ethical Considerations: All participants will provide informed consent.
- Study Design: A within-subjects experimental design will be employed, where each participant experiences all conditions. This minimizes the variance due to individual differences and increases the statistical power of the experiment.
- Counterbalancing: The order of condition presentation (C1, C2, C3) will be counterbalanced across participants using a Latin Square design to mitigate learning and fatigue effects.

#### 3.2.1 Experimental conditions (control groups)

To isolate the effect of intelligent proactivity, each participant interacted with the robot under three control conditions, as shown in Table 4.

**TABLE 4:** Experimental condition design.

Cond.	Name	Description of robot action	Purpose of comparison
C1	*Explicit reactive*	The robot remains static. Humans must explicitly request the next part.	Establishes the baseline for traditional, non-intelligent HRC.
C2	*Intelligent reactive*	The robot uses vision without prediction to recognize when a human has completed a subtask. After completion, it delivers the next part of the text.	Isolates the benefit of proactivity (C3) over reactivity (C2); measures waiting time cost.
C3	*Proactive framework (proposed)*	The robot employs the full framework (Cognitive Model + DRL SAC). It anticipates subtask completion and moves to deliver the next part *before* the human completes the current action.	Tests the full hypothesis: whether intention-based proactivity improves efficiency, fluency, and human factors.

These experimental conditions were designed to isolate the incremental contributions of intention prediction and proactive control, enabling a fair comparison with reactive baselines in terms of fluency, workload, and safety.

### 3.3 Key evaluation metrics

The evaluation will be holistic, combining both objective and subjective metrics. These conditions were defined to isolate the effect of intention-based proactivity once empirical validation was conducted.

#### 3.3.1 Cognitive model performance - offline evaluation

- Prediction Accuracy: Measured using accuracy and F1-score for intention classification $\left(I_{\text{pred}}\right)$ against ground-truth labels:

$$F_{1}=\frac{2\cdot \text{Precision}\cdot \text{Recall}}{\text{Precision}+\text{Recall}}.$$ (20)

- Prediction Horizon (H): Average time in seconds by which the system predicts an intention before the human initiates the physical action:

$$H=t_{\text{start}}-t_{\text{pred}}.$$ (21)

#### 3.3.2 Task performance and efficiency - objective metrics

- Task Completion Time (TCT): Total time required to complete the assembly.
- Human Idle Time $\left(t_{\text{idle\_h}}\right)$ : Time the human spends waiting for the robot.
- Robot Idle Time $\left(t_{\text{idle\_r}}\right)$ : Time the robot spends waiting for the human.

#### 3.3.3 Safety and fluency - objective metrics

- Number of Stops/Collisions: Count of safety-related events.
- Minimum Human-Robot Distance $\left(d_{\text{min}}\right)$ : Minimum maintained distance during interaction.
- Motion Smoothness (Jerk): Measured as the root mean square jerk $\left(J_{\text{rms}}\right)$ of the end-effector trajectory. Lower values indicate smoother and less erratic motion.

$$J_{\text{rms}}=\sqrt{\frac{1}{T}\int_{0}^{T}\|{\dddot{p}}_{ef}\left(t\right){\|}^{2}\;dt}.$$ (22)

#### 3.3.4 Human factors - subjective metrics

- Perceived Workload: Measured using the NASA-TLX questionnaire, which evaluates six dimensions: Mental Demand, Physical Demand, Temporal Demand, Effort, Performance and Frustration (Hart and Staveland, 1988).
- Trust and Perceived Safety: Evaluated using standardized questionnaires with Likert scales to assess human trust and perceived safety.
- Perceived Fluency: Subjective rating of how “natural” or “fluid” the collaboration felt.

### 3.4 Result hypotheses

The following quantitative hypotheses are expected to be validated by comparing the C1 (Explicit Reactive), C2 (Intelligent Reactive), and C3 (Proactive) conditions:

- H1 (Efficiency): Task Completion Time (TCT) will be significantly lower in C3 than in C2, and in C2 than in C1: $\left(TCT_{C3}<TCT_{C2}<TCT_{C1}\right)$ .
- H2 (Objective Fluency): Human Idle Time $\left(t_{\text{idle\_h}}\right)$ will be drastically reduced approaching zero in C3 compared to C1 and C2.
- H3 (Cognitive Load): NASA-TLX particularly Mental Demand and Frustration scores will be significantly lower in C3 than in C1 and C2.
- H4 (Trust and Perception): Trust and Perceived Safety scores will be significantly higher in C3, where participants perceive the robot as a more competent “teammate.”
- H5 (Model Accuracy): The multimodal cognitive model (C3) is expected to achieve a statistically significant improvement in intention prediction accuracy and prediction horizon compared to unimodal baselines.

#### 3.4.1 Statistical validation methods

To evaluate the hypotheses, a rigorous statistical analysis based on a within-subject design (N=30) will be employed. A significance level of α=0.05 will be established for all the tests.

- Validation of H1 and H2 (Quantitative Metrics). Objective performance metrics (TCT, $t_{\text{idle\_h}}$ ) will be analyzed using a one-way repeated-measures ANOVA (RM-ANOVA), with the factor *Condition* (C1, C2, C3) as within-subject variable. The sphericity assumption will be verified using Mauchly’s test; if it is violated, the Greenhouse-Geisser correction will be applied. If the RM-ANOVA yields significant results, *post hoc* pairwise comparisons will be conducted using paired t -tests with Bonferroni correction to control the Type I error rate.
- Validation of H3 and H4 (Subjective/Ordinal Metrics). For subjective data collected from Likert-scale questionnaires, which are ordinal in nature, the non-parametric Friedman test will be applied. If a statistically significant difference is detected among the three conditions, *post hoc* pairwise comparisons (C1-C2, C2-C3, C1-C3) will be performed using the Wilcoxon signed-rank test, again applying a Bonferroni correction.
- Validation of H5 (Model Performance). H5 will be validated offline using a retained test dataset. The F1-score and the prediction horizon of the proposed multimodal model will be compared against a unimodal baseline. A paired t -test or Wilcoxon signed-rank test, depending on the normality of the cross-validation residuals, will be used to determine whether the observed improvement in performance is statistically significant.

## 4 Discussion

This study provides a formally grounded methodological framework that bridges multimodal intention prediction and proactive control in HRC. However, its empirical validation is still pending and will require careful consideration of the variability in human behavior, sensor noise, and industrial constraints. Future work will focus on implementing the protocol in real assembly cells, extending the intention taxonomy, and assessing generalization across tasks and operators.

A potential concern regarding the proposed framework is its architectural complexity. However, this design is motivated by the need to explicitly couple perception, cognition, and control in proactive collaboration scenarios. Future ablation studies are planned to quantify the contributions of each module.

If these hypotheses are empirically confirmed in future studies, the proposed framework would provide evidence that the formal integration of intention prediction or CM into the control loop enables synergistic collaboration. While previous studies have focused on motion prediction (Zhou et al., 2024) or gaze-based intention inference (Di Stefano et al., 2025) in isolation, this framework unifies these components within a closed perception-cognition-action cycle.

A collaborative robot capable of predicting both the operator’s intention and motion is expected to provide proactive assistance. For instance, if the robot anticipates that the operator will soon require part B and detects that the operator’s hand is moving toward that part within approximately 1.2 s, it can retrieve and pre-position the component in advance. This predictive capability stands in contrast to that of a reactive robot (C2), which performs an action only after the human has completed the preceding task.

Furthermore, incorporating force-torque data (Zhao, 2025; Sliwowski et al., 2025) and fluency reward $\left(r_{\text{flu}}\right)$ into the DRL controller will allow the robot to manage contact-rich tasks more intelligently and smoothly, adapting its behavior to differentiate intentional or guided from accidental contacts.

The proposed framework and protocol are explicitly designed to address key research gaps: (1) integrating task context (objects, state $S_{\text{task}}$ ) is expected to outperform human motion prediction in isolation (Gómez-Izquierdo et al., 2025), and (2) the online connection between CM and DM bridges cognition and action, demonstrating the effective transfer of inferred intention into adaptive control.

Prior to physical deployment, the proactive controller will be validated in a high-fidelity simulation environment such as CoppeliaSim. This virtual stage allows for the verification of the SAC commands, ensuring that the generated velocity profiles are within the robot’s kinematic limits and that proactive trajectories do not violate safety boundaries under simulated human stochasticity.

### 4.1 Extended challenges: safety, ethics, and industrial transfer

Despite the robustness of the proposed methodology, several challenges were anticipated. These represent inherent limitations and key discussion points for real-world industrial transfer.

To ensure industrial stability, the system architecture accounts for a total control loop latency of approximately 80–100 ms , which includes sensor data acquisition ∼33 ms , CNN-LSTM-Transformer inference ∼40 ms , and ROS 2 communication overhead.

Robustness against sensor noise and uncertainties will be addressed by implementing extended Kalman filters for state estimation and utilizing the entropy-regularized exploration of the SAC algorithm, which prevents the policy from over-fitting to noisy observation signals.

#### 4.1.1 Data generalization and the sim-to-real challenge

Deep learning models require large volumes of labeled data to train. A major challenge will be ensuring that the model generalizes to new users or to slight variations in the assembly task (Adebayo et al., 2024; Sliwowski et al., 2025).

Moreover, training the DRL controller directly on hardware is slow and potentially unsafe; therefore, training primarily occurs in simulation. The simulation-to-reality transfer gap remains an open issue in robotics research (Yuan et al., 2022). Husakovic et al. (2025) reported 100% success in simulation but only 80% on real hardware, clearly illustrating this difficulty.

To mitigate this gap, advanced techniques such as *Domain Randomization* and *Curriculum Learning* will be employed. In addition, “semi-virtual environments” can be used, combining a real robot with simulated sensors or obstacles to introduce controlled randomness while maintaining interaction with physical hardware.

#### 4.1.2 Regulatory safety, proactivity, and trust

During DRL training, exploration can lead to unsafe behaviors. In addition to training, industrial deployment must comply with strict safety standards. These include ISO 10218:2025, which integrates and updates the directives of ISO/TS 15066. The standards require a detailed risk assessment for each specific application and define four modes of collaboration, of which Power and Force Limiting (PFL) is the only one that allows physical contact.

A conceptual challenge is that current safety regulations are inherently reactive, whereas the proposed framework is proactive. Robots that move anticipatorily introduce new safety paradigms that current reactive norms do not fully address.

However, prior research suggests that proactive systems, when properly designed, can foster greater trust and perceived intelligence than purely reactive systems, which is fundamental for user acceptance and regulatory adaptation.

#### 4.1.3 Ethical implications and industrial adoption

From an Industry 5.0 perspective, intention prediction acts as a human-in-the-loop communication channel rather than an automated mechanism. By anticipating needs while preserving human authority over task execution, the framework aligns with human-centric manufacturing principles, complementing recent discussions on sustainable and ethical HRC systems (Dhanda et al., 2025; Cai et al., 2026).

The industrial adoption of cognitive cobots faces significant ethical challenges. The introduction of experimental AI into the workplace has been described as a “social experiment” that must be governed by the principles of beneficence, justice, and responsibility.

A central concern is human autonomy: a system that predicts intentions must not coerce or manipulate the worker and should always operate under the supervision of a human. In addition, the use of multimodal sensors, which are necessary for intention prediction, raises serious concerns regarding privacy and data protection for workers, factors that negatively affect their willingness to adopt such systems.

Ultimately, the success of the system depends on the trust of the users. Prior work has shown that “trust and safety” are the dominant variables that influence a human operator’s intention to use or rely on a robot. Therefore, the design objective should be human-centered, focusing not only on productivity metrics but also on ensuring that technology augments human capabilities rather than displacing them.

#### 4.1.4 Explainable artificial intelligence (XAI)

Deep learning and DRL models are inherently “black boxes.” This presents a challenge for operator trust and safety certification (Pietrantoni et al., 2024). Although the proposed system may be effective, its lack of explainability remains a recognized limitation. Recent reviews argue that achieving safe and deployable human–robot collaboration often requires combining complementary algorithmic components, such as perception, prediction, control, and safety supervision, rather than relying on a single technique (Lefranc et al., 2025).

Future work should explore explainable AI techniques, such as attention visualization, *post hoc* interpretability methods, and policy attribution, to make proactive decisions more transparent and auditable, thereby aligning cognitive robotics with ethical and regulatory requirements for Industry 5.0.

## 5 Conclusion

This study presents a comprehensive and methodologically detailed framework proposal designed to overcome the predominantly reactive nature of current collaborative robotics. This advances toward a proactive and cognitive collaboration model aligned with the principles of Industry 5.0.

The main contribution of this proposal lies not only in the conceptualization of a system but also in its rigorous mathematical formalization. By defining the collaboration task as a Markov Decision Process, where the state vector is augmented with the predicted human intention $P\left(I_{t+k}\right)$ , a methodological bridge is established between cognition and action. This formalization, combined with a reward function tailored to synergy and fluency, enables the robot to learn proactively.

Furthermore, this study presents a rigorous experimental validation protocol grounded in a within-subject design and supported by a comprehensive set of evaluation metrics. These include objective performance indicators such as efficiency, safety, and interaction fluency, as well as subjective human-centered factors encompassing perceived workload measured using the NASA–TLX, user trust, and perceived fluency in collaboration.

In summary, the proposed framework and protocol offer a reproducible blueprint for the design, training, and evaluation of proactive cobots in Industry 5.0 scenarios. By explicitly coupling perception, cognition, and control, this study aims to reduce the gap between theoretical HRC architectures and implementation-ready solutions for industrial assembly.

In future work, the framework will be implemented on the uFactory Lite 6 platform to empirically test the hypotheses under the proposed protocol. Although empirical validation is beyond the scope of this manuscript, the present contribution provides a rigorous methodological foundation for the future integration of artificial intelligence and cognitive robotics within human-centered manufacturing environments.

## Data Availability

The original contributions presented in the study are included in the article/supplementary material, further inquiries can be directed to the corresponding author.
